# Factors Associated With Severe Dengue During the 2019 Outbreak in Southern Colombia: Implications for Hyperendemic Regions

**DOI:** 10.1155/jotm/2084096

**Published:** 2026-09-22

**Authors:** Luis F. Oliveros, Camilo Eduardo Rivera, María Clemencia Rojas, Carlos F. Narváez

**Affiliations:** ^1^ Secretaría de Salud Departamental, Gobernación del Huila, Neiva 41001, Huila, Colombia; ^2^ Programa de Enfermería, Facultad de Ciencias de la Salud, Universidad Surcolombiana, Neiva 41001, Huila, Colombia, usco.edu.co; ^3^ Dirección Laboratorio de Salud Pública, Secretaría de Salud Departamental, Gobernación del Huila, Neiva 41001, Huila, Colombia; ^4^ División de Inmunología, Programa de Medicina, Facultad de Ciencias de la Salud, Universidad Surcolombiana, Postgrado Médico de Pediatría, Hospital Universitario de Neiva, Neiva 41001, Huila, Colombia, hospitaluniversitarioneiva.com.co

**Keywords:** bleeding, children, Colombia, dengue, fluid accumulation, organ involvement, outbreak, severe dengue, thrombocytopenia

## Abstract

**Introduction:**

In 2019, Colombia reported over 127,000 dengue virus (DENV) infections, with Huila accounting for 20% of the national total of severe dengue (SD) cases. This highlights the need to identify the local determinants of severity.

**Methods:**

We examined 5494 dengue and 251 SD cases with laboratory confirmation reported in Huila, southern Colombia, in 2019. Multiplex RT‐qPCR was performed on 571 samples to determine viral serotypes. Multivariate logistic regression adjusted for age, sex, and comorbidities assessed epidemiological, clinical, and virological factors associated with SD.

**Results:**

SD manifested as vascular leakage with or without shock (76%), organ involvement (13%), severe bleeding (2.7%), and multiple severe manifestations in 8.3%. A trend toward higher frequency of severity among vulnerable patients in the subsidized health insurance system was observed. DENV‐1 was the most prevalent serotype, but DENV‐2 was associated with severity. Age < 5 years (OR = 4; 95% CI: 2.3–6.8), 5–15 years (OR = 1.9; 95% CI: 1.1–3.1), diarrhea (OR = 2.1; 95% CI: 1.5–2.9), vomiting (OR = 1.8; 95% CI: 1.2–2.2), abdominal pain (OR = 3.2; 95% CI: 2.3–4.6), hepatomegaly (OR = 4.7; 95% CI: 3.4–6.7), platelet count < 100,000/μL (OR = 7.2; 95% CI: 5.2–10.1), and fluid accumulation (OR = 10.3; 95% CI: 6–17.8), were the main factors associated with SD.

**Conclusion:**

Young age, gastrointestinal manifestations, hepatomegaly, thrombocytopenia, fluid accumulation, and DENV‐2 infection were associated with SD during the 2019 epidemic in southern Colombia.

## 1. Introduction

Dengue is an acute viral disease transmitted by mosquitoes of the genus *Aedes*, clinically characterized by fever, headache, myalgia, arthralgia, and rash (exanthema). Severe forms of the infection, including plasma leakage, severe bleeding, and organ dysfunction, account for about 5% of hospitalized cases and can be potentially fatal [1]. Currently, there is no specific antiviral treatment; however, since 2026, the Qdenga vaccine, a tetravalent chimeric live attenuated virus formulation, has been available in Colombia to priority populations, such as 9‐year‐old children or those enrolled in the fourth grade of primary school.

The global incidence and mortality associated with dengue have steadily increased over the last few decades. Between 1990 and 2017, the number of dengue virus (DENV) infections increased fivefold, and the case fatality doubled in the same period [2, 3]. In 2019, the Americas experienced the second‐largest recorded dengue epidemic, with more than 3 million symptomatic cases; Colombia reported approximately 127,000, including over 1400 severe cases, making it one of the most affected countries in the region [4, 5].

Between 2010 and 2020, Colombia recorded more than 950,000 cases of dengue, of which 24,000 were classified as severe, ranking second in Latin America in disease burden after Brazil (INS, Colombia, weekly epidemiological bulletins, Week 53 from 2010 to 2020)[6]. In this context, the department of Huila—located in the south of the country and with a population of approximately 1.15 million inhabitants—has historically been classified as a hyperendemic zone for emerging and reemerging arboviruses [7–9]. In the last decade, more than 57,000 dengue cases have been reported in this region, including 1623 severe cases, with Neiva, its capital city, among the cities that most frequently report cases nationally.

The 2019 epidemic had a particularly severe impact in Huila, with more than 11,000 suspected and 5700 confirmed dengue cases, placing the department third nationally in the number of cases [4]. In addition, it had a dengue mortality rate twice the national average and accounted for 20% of all severe dengue (SD) cases, the highest in the country. The observed prevalence of SD was notably higher than that reported nationally for Colombia in 2019, underscoring the disproportionate severity of the epidemic in the Huila department and justifying the focus on this region to identify local factors for severe disease [4]. Notably, during this epidemic, we also reported pediatric cases of hemophagocytic lymphohistiocytosis, a rare but lethal manifestation of SD [10], broadening the known clinical spectrum and reinforcing the need for detailed characterization of these outbreaks at the regional level.

This study aimed to identify specific epidemiological, clinical, and virological factors—including age, clinical warning signs, laboratory parameters, and DENV serotype—associated with SD during the 2019 epidemic in the Huila Department. Our aim is not to identify new predictors, but to quantify how established WHO 2009 warning signs and key laboratory markers relate to SD in a large, predominantly pediatric cohort from a hyperendemic Colombian department that contributed 20% of the country’s severe cases in 2019. To do that, this study analyzed 5745 confirmed dengue cases, including 251 cases of SD, reported during the 2019 epidemic in this hyperendemic area.

## 2. Methods

### 2.1. Study Design and Population

**FIGURE 1:**
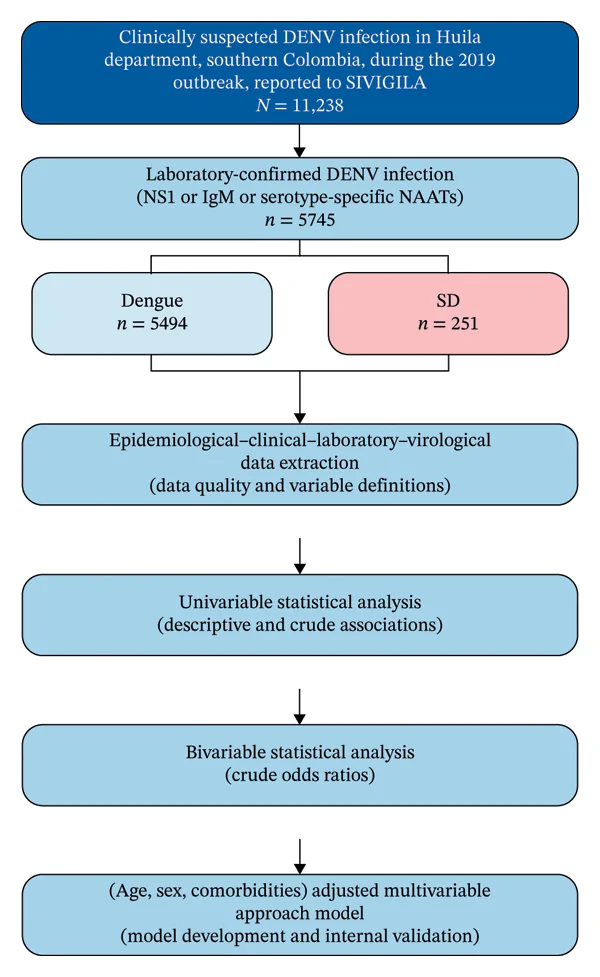
Study population and analytic workflow. The diagram summarizes the selection of dengue and SD cases from SIVIGILA during the 2019 outbreak in Huila and outlines the main analytical steps, from data extraction and descriptive analyses through bivariable and multivariable modeling, to the identification of factors associated with SD. SIVIGILA: sistema de vigilancia en salud pública. NAAT: nucleic acid amplification test. Figure created in BioRender under license FQ292T4XJF.

An observational, analytical study was conducted using a retrospective cross‐sectional analysis of symptomatic dengue cases reported to the National Epidemiological Surveillance System (SIVIGILA) between January 1 and December 31, 2019. For practical reasons and because the study aimed to identify factors associated with SD, the dengue without warning signs (DNS) and dengue with warning signs (DWS) subgroups (globally named the “dengue” group) were analyzed together and compared with the SD group. Cases with codes “210” (“dengue”) and “220” (“severe dengue”) from the department of Huila were included. Not all suspected dengue cases reported to SIVIGILA underwent laboratory confirmation. In accordance with national guidelines and local resource constraints, diagnostic tests are typically requested for a subset of suspected cases, prioritizing patients with compatible symptoms and/or warning signs or those evaluated in higher level facilities. For this study, we included only cases with laboratory confirmation for the main cohort of interest, while recognizing that the broader surveillance system also captured untested clinically suspected cases. All laboratory‐confirmed dengue cases (51.1% of the total suspected cases) followed the Colombian surveillance case definition and included those with acute serum‐positive IgM (most cases), NS1 antigen, nucleic acid amplification tests (NAATs), or viral isolation. For the detection of DENV‐specific IgM or NS1 antigen in acute serum, the commercial IgM Serion ELISA Classic (Virion Serion, Cat. ESR114M) and the Dengue Detect NS1 ELISA kits (InBios, Cat. DNS1‐1, Seattle, WA, USA) were used. In a fraction of patients, molecular methods or viral isolation were used according to the guidelines of the Instituto Nacional de Salud (INS) of Colombia. The complete study design is illustrated in Figure 1.

Because data were obtained retrospectively from a surveillance registry at the time of notification, we could not determine the exact temporal sequence between the onset of individual clinical signs or laboratory abnormalities and the classification of SD. Therefore, all associations should be interpreted as cross‐sectional correlates rather than longitudinal predictors of disease progression.

### 2.2. Data Quality and Completeness

In Colombia, dengue is under mandatory surveillance through the SIVIGILA. Suspected or confirmed dengue cases seen in public and private health facilities are reported using a standardized notification form, which is completed by trained clinical or epidemiologic staff based on information in the medical record and available laboratory results. These case reports are submitted electronically to municipal and departmental health authorities, where they undergo basic validation and consolidation into the SIVIGILA database, which served as the data source for this study.

All dengue notifications received by the Secretaría de Salud Departamental del Huila were subject to routine validation checks within SIVIGILA, including range verification, internal consistency assessment (e.g., consistency between age, clinical classification, and outcome), and duplication of reports for the same patient. We excluded records that lacked laboratory confirmation or showed inconsistent classifications that could not be resolved after data cleaning. For this study, we performed an additional data‐cleaning step to exclude records with duplicate identifiers or implausible values and to characterize the extent of missing data for key variables (clinical warning signs, laboratory parameters, and severity classification). Analyses were conducted on complete‐case data for each model. Potential underreporting and nondifferential misclassification inherent to mandatory surveillance systems are acknowledged as limitations in the Discussion section.

### 2.3. Operational Definitions

Age was analyzed as a categorical variable to capture nonlinear risk associations. The categories (< 5, 5–15, 16–30, 31–60, > 60 years) were chosen based on established pediatric age brackets and to reflect broad adult life stages. “Children” were defined as patients aged 0–15 years, consistent with the youngest age categories (younger than 5 years and 5–15 years) in the models. We acknowledge that the adult categories, particularly the 31–60 age group, encompass a wide range of physiological states. This grouping was necessary to maintain sufficient statistical power for analysis, but may mask more nuanced, age‐specific risk relationships within the adult population. For the remaining variables, the following criteria were uniformly applied in case adjudication using available clinical records, ensuring reliability and minimizing endpoint misclassification. Clinical warning signs were defined according to the WHO 2009 guidelines and the standardized case report forms of the SIVIGILA as follows: (i) Abdominal pain: documented in the medical record. While the SIVIGILA form does not specify intensity or progression, its classification as a “warning sign” in this context implies pain reported as severe or persistent enough to raise clinical concern, distinct from mild, transient discomfort. (ii) Persistent vomiting: recorded as present based on the clinical assessment documented in the chart. This was defined as three or more episodes of vomiting within 24 h. (iii) Hepatomegaly: defined as a palpable liver below the costal margin upon physical examination, as documented by a healthcare provider in the patient’s clinical record. Specific measurements (e.g., in centimeters) were not standardized in the surveillance dataset. (iv) Fluid accumulation: the presence of clinical ascites, pleural effusion, or pericardial effusion detected through physical examination, imaging (e.g., ultrasound, radiography), or both. (v) Mucosal bleeding: Epistaxis, gingival bleeding, melena, hematemesis, or other clinically significant hemorrhage documented in the record. The use of these standardized surveillance definitions ensures consistency in reporting across the department, although it is acknowledged that some granularity of clinical detail is inherent to this methodology.

All endpoints and SD classifications were operationalized using the 2009 WHO criteria and national clinical guidelines as follows: (i) Hypotension was defined as a systolic blood pressure below the 5^th^ percentile for age (children), < 90 mmHg for adults, the presence of narrow pulse pressure (< 20 mmHg), or clinical signs of poor perfusion. (ii) Severe fluid leakage was considered present if shock or fluid accumulation resulted in respiratory distress. (iii) Shock based on clinical diagnosis was defined as hypotension, cold/clammy extremities, weak, rapid pulse, and delayed capillary filling. (iv) Severe bleeding was determined by clinical judgment and included gastrointestinal bleeding, persistent mucosal bleeding, or any bleeding that led to hemodynamic compromise. (v) Severe organ involvement included evidence of hepatitis (AST or ALT ≥ 1000 IU/L), impaired consciousness (encephalopathy), myocarditis (clinical or biochemical markers), or acute renal impairment not explained by other causes.

Below, in the multivariable model, we refer to variables such as fluid accumulation, increased hematocrit, and hypotension as “clinical markers of severity” rather than “risk factors,” given their close overlap with the WHO criteria used to define SD.

Because no dengue vaccine was included in the Colombian National Immunization Program during 2019 and population coverage was negligible, vaccination status was not systematically recorded in SIVIGILA and was not analyzed.

### 2.4. Sample Selection for Serotyping

Serotyping was performed on a convenience sample of 571 cases selected based on the availability of sufficient residual plasma in the laboratory archive after routine diagnostic testing. Although probability sampling was not feasible, the selection process was not based on clinical or demographic characteristics. To assess the potential for selection bias, we compared some demographic and clinical profiles of the typed subcohort with those of the larger cohort of nontyped confirmed dengue cases (Supporting Table 1). Despite this severity‐based enrichment, the serotyped subgroup was broadly similar to the overall cohort in terms of age distribution, sex, and geographic origin (Supporting Table 1), although the proportion of SD was higher among typed cases, which constitutes an advantage as the number of total SD cases was markedly lower than that of dengue cases, supporting its use for exploratory description of circulating serotypes. Globally, the data support the representativeness of the subsample for describing the serotype distribution in the overall population.

### 2.5. NAATs for Viral Serotype Identification

Nested RT‐PCR (115 tests, 20%) or multiplex RT‐qPCR (456 tests, 80%) (collectively referred to as NAATs) was performed on 571 plasma samples from confirmed cases to identify the infecting DENV serotype, following previously described protocols [11, 12]. Total RNA was extracted automatically from 200 μL of plasma using the MagMax Viral/Pathogen II kit (Thermo Fisher Scientific, Cat No A48383) and KingFisher Flex automated kit (Cat No 5400610) as previously reported [13].

For nested RT‐PCR, a common 512‐bp fragment was amplified, and then serotype‐specific primers were used to amplify 312‐bp (DENV‐1), 110‐bp (DENV‐2), 280‐bp (DENV‐3), and 332‐bp (DENV‐4) fragments. The products were visualized on a 2% agarose gel, stained with ethidium bromide, and analyzed in a digital documentation system (GelDoc, Bio‐Rad).

Multiplex RT‐qPCR was performed on a QuantStudio 5 thermal cycler (Thermo Fisher Scientific, Cat No A28134), using the SuperScript III Platinum One‐Step qRT‐PCR kit (Invitrogen, Cat No 11732–088). Positive controls (supernatant from cultures infected with each serotype), negative controls (uninfected cultures), and no‐template controls (NTC) were included in each assay.

### 2.6. Statistical Analysis

Statistical analyses were performed using Stata v.17 (StataCorp LLC, College Station, TX, USA). The variables were grouped as follows: (i) sociodemographic: age, sex, area of residence, health system affiliation, socioeconomic stratum, and membership in special populations (ethnic minority, physical disability, displacement due to armed conflict, demobilized member of an illegal armed group, among others); (ii) clinical: fever, headache, abdominal pain, vomiting, diarrhea, drowsiness, hypotension, and hemodynamic instability; (iii) laboratory: elevated hematocrit and thrombocytopenia (< 100,000/mm^3^); and (iv) clinical classification: dengue ([DNS] plus [DWS]) or SD. Analyses were conducted using complete‐case data for each model, and the proportion of missing values for key variables was summarized to characterize the extent of missing data.

Categorical variables (mostly dichotomous) are presented as absolute and relative frequencies (%). Continuous variables are summarized as medians and interquartile ranges, because the Shapiro–Wilk test indicated nonnormal distributions. Comparisons between the dengue and SD groups were performed using the Mann–Whitney U test. Comparisons of categorical variables were performed using the chi‐square test when all expected cell counts were ≥ 5 and Fisher’s exact test when any expected cell count was < 5, as specified; although most frequency analyses used the chi‐square test, Fisher’s exact test was applied.

To quantify the association between each candidate factor and SD, we first fitted bivariate logistic regression models and estimated crude odds ratios (ORs) with 95% confidence intervals (CIs). We then constructed a multivariable logistic regression model, starting from a full model including variables selected a priori for their clinical/epidemiological relevance or showing a bivariate association with SD at *p* < 0.05, and applied backward stepwise elimination [14]. Multivariable models included a core set of adjustment variables and other clinically relevant variables specified a priori, while bivariate associations were used only to refine the inclusion of secondary covariates rather than to select variables solely on the basis of univariable *p* values [15]. Thus, age, sex, and presence of any documented comorbidity were forced into the final model, regardless of statistical significance, to control for basic demographic and health status confounding. Comorbidities included hypertension, diabetes mellitus, chronic cardiovascular disease, chronic renal disease, asthma, and other chronic respiratory conditions, as recorded in SIVIGILA. We assessed multicollinearity by examining variance inflation factors and evaluated overall model fit using standard diagnostics, including the Hosmer–Lemeshow goodness‐of‐fit test; no major violations of model assumptions were detected. All tests were two‐sided, and *p* < 0.05 was considered statistically significant.

Because only limited information was available on key prognostic domains (e.g., serological status, viral load, and time from symptom onset to consultation), our models are adjusted only for basic demographic and comorbidity structure. In addition, some clinical and laboratory variables in the models partially overlap with the WHO 2009 criteria used to classify SD; accordingly, the estimated ORs should be interpreted as measures of association and markers of severe disease, not as independent causal predictors or direct estimates of risk or prevalence ratios.

## 3. Results

### 3.1. Demographic and Epidemiological Characteristics

During the 2019 dengue epidemic in Huila, 11,238 clinically suspected cases were reported, of which 5745 were laboratory confirmed. Of all confirmed cases, the majority (94.4%) were diagnosed by IgM‐DENV testing. As shown in Figure 1 and according to the WHO 2009 criteria, 5494 cases were classified as dengue (which included DNS and DWS), and 251 cases as SD (Table 1). In our setting, 4150 patients were under 15 years (Table 1), 4384 were under 18 years, and 1361 were adults (≥ 18 years, not shown), confirming that the 2019 epidemic in Huila predominantly affected children and adolescents. Patients with SD were significantly younger (median 6 vs. 9 years; *p* < 0.001, Table 1), with 33.4% of SD in those younger than 5 years (see Table2). No significant differences were found in sex, area of residence, type of health insurance, or membership in special populations. However, there was a trend toward a higher proportion of severe cases among patients with subsidized health insurance (*p* = 0.062, Table 1). Overall, these findings show that 51% of dengue cases reported in Huila were laboratory‐confirmed and that 4.3% met the criteria for SD, a condition that predominantly occurred in younger children.

**TABLE 1:** Epidemiological and social characteristics of the patients with dengue and SD during the 2019 outbreak in Huila, Colombia.

Characteristics	Dengue (*n* = 5494)	SD (*n* = 251)	*p* value
Age, years, median (range)	9 (1–90)	6 (1–71)	< 0.001^a^

Children, ≤ 15 years old, *n* (%)	3932 (71.6)	218 (86.9)	< 0.0001^b^

Female/male, *n* (%)	2811 (51.2)/2683 (48.8)	129 (51.4)/122 (48.6)	0.949^b^

Urban residence, *n* (%)	4792 (87.3)	213 (84.9)	0.289^b^
Rural residence, *n* (%)	702 (12.7)	38 (15.4)	

Contributory health insurance, *n* (%)	2044 (37.2)	106 (42.2)	0.062^b^
Subsidized health insurance, *n* (%)	3231 (58.8)	131 (52.2)	

No health insurance, *n* (%)	219 (4.0)	14 (5.6)	0.210^b^
Special population^∗^, *n* (%)	85 (1.5)	6 (2.3)	0.293^b^

*Note:* SD: severe dengue according to WHO 2009 guidelines.

^a,b^ *p* value of the Mann–Whitney and chi‐square tests are presented.

^∗^Special population: migrant individuals, population displaced by armed conflict, inmates, or individuals reinserted from any illegal group.

**TABLE 2:** Factors associated (crude OR) in the study population.

Characteristics	Dengue (*n* = 5494)	SD (*n* = 251)	Crude OR^∗^	95% CI	*p* value
*Age*					
Younger than 5 years	1166 (21.2)	84 (33.4)	3.05	1.89–4.91	< 0.0001
5–15 years	2766 (50.3)	134 (53.3)	2.05	1.30–3.24	0.002
16–30 years	931 (16.9)	22 (8.7)	1.0 Ref		
31–60 years	513 (9.3)	8 (3.2)	0.66	0.29–1.49	0.318
> 60 years	118 (2.1)	3 (1.2)	NE^∗^		

*Gender*					
Female	2811 (51.1)	129 (51.3)	1.0 Ref		
Male	2683 (48.8)	122 (48.6)	0.99	0.77–1.28	0.943

*Residence area*					
Urban	4792 (87.2)	213 (84.6)	0.82	0.58–1.17	0.275
Rural	702 (12.7)	38 (15.1)	1.0 Ref		

*Social security*					
Contributory health insurance	2044 (37.2)	106 (42,2)	1.0 Ref		
Subsidized health insurance	3231 (58.8)	131 (52.2)	0.76	0.59–0.99	0.038
No health insurance	219 (4.0)	14 (5.6)	1.42	0.82–2.48	0.214
Special population	85 (1.5)	6 (2.4)	1.56	0.67–3.60	0.299

*Warning signs*					
Abdominal pain	1597 (29.1)	189 (75.3)	7.44	5.55–9.97	< 0.0001
Persistent vomiting	1417 (25.8)	155 (61.8)	4.65	3.58–6.03	< 0.0001
Thrombocytopenia < 100,000 cell/mm^3^	1055 (19.2)	191 (76.1)	13.39	9.95–18.04	< 0.0001
Diarrhea	844 (15.4)	104 (41.4)	3.90	3.0–5.06	< 0.0001
Somnolence or irritability	256 (4.7)	51 (20.3)	5.22	3.74–7.27	< 0.0001
Mucosal bleeding	282 (5.1)	37 (14.7)	3.20	2.21–4.62	< 0.0001
Hepatomegaly	241 (4.4)	100 (39.8)	14.43	10.87–19.17	< 0.0001
Increased hematocrit	168 (3.1)	40 (15.9)	6.01	4.15–8.71	< 0.0001
Hypotension	86 (1.6)	30 (11.9)	8.54	5.52–13.21	< 0.0001
Fluid accumulation	35 (0.6)	51 (20.3)	39.77	25.29–62.55	< 0.0001
Hypothermia	23 (0.4)	4 (1.5)	NE^∗^		

*Note:* SD: severe dengue according to the WHO 2009 guidelines. For social security, contributory health insurance was used as the reference category; odds ratios for the other categories (e.g., subsidized, uninsured) are estimated relative to this group.

^∗^NE: not estimable for a low number of patients.

### 3.2. Clinical Characteristics of the DENV‐Infected Populations

Warning signs were present in 2647 (48.2%) patients classified as dengue. In contrast to the dengue group, patients with SD had a higher frequency of all clinical signs and laboratory parameters evaluated (Table 3). Among the most common signs in the severe group were abdominal pain (75.3%), persistent vomiting (61.8%), diarrhea (41.4%), somnolence/irritability (20.3%), and thrombocytopenia < 100,000/mm^3^ (76.1%), all of which differed significantly from those in the dengue group (*p* < 0.001, Mann–Whitney test). Likewise, hepatomegaly (39.8%) was significantly more frequent in the SD group.

**TABLE 3:** Clinical and virological characteristics of the patients with dengue and SD during the 2019 outbreak in Huila, Colombia.

Variable	Dengue (*n* = 5494)	SD (*n* = 251)	*p* value
Warning signs, *n* (%)	2647 (48.2)	251 (100%)	< 0.001
Abdominal pain	1597 (29.1)	189 (75.3)	< 0.001
Persistent vomiting	1417 (25.8)	155 (61.8)	< 0.001
Platelets < 100,000 cells/mm^3^	1055 (19.2)	191 (76.1)	< 0.001
Diarrhea	844 (15.4)	104 (41.4)	< 0.001
Somnolence or irritability	256 (4.7)	51 (20.3)	< 0.001
Mucosal bleeding	282 (5.1)	37 (14.7)	< 0.001
Hepatomegaly	241 (4.4)	100 (39.8)	< 0.001
Increased hematocrit	168 (3.1)	40 (15.9)	< 0.001
Hypotension	86 (1.6)	30 (12.0)	< 0.001
Fluid accumulation	35 (0.6)	51 (20.3)	< 0.001
Hypothermia	23 (0.4)	4 (1.5)	0.028

*Severe dengue signs, n (%)*			
Severe plasma leakage with shock	NA	106 (42.2)	—
Severe plasma leakage	NA	84 (33.4)	—
Severe organ involvement	NA	33 (13.1)	—
Severe bleeding	NA	7 (2.7)	—
Patients with two or more signs of SD	NA	21 (8.3)	—

*Virological tests, n (%)*			
DENV1‐4 NAATs performed^a^	513 (9.4)	58 (23.1)	< 0.0001
Typeable samples^b^	275 (53.6)	23 (40)	0.2696
DENV‐1^c^	235 (85.4)	10 (43.5)	0.0007
DENV‐2^c^	40 (14.6)	13 (56.5)	
DENV‐3	0	0	—
DENV‐4	0	0	—
No typeable samples^d^	238 (46.4%)	35 (60%)	0.2902

*Note:* SD: severe dengue according to the WHO 2009 guidelines. NA: not applicable to the variable. RT‐PCR DENV1‐4: retrotranscribed‐quantitative polymerase chain reaction for DENV in multiplex format. Chi‐square and Fisher’s exact tests were used.

^a^Number (%) of performed tests relative to each of the “Dengue” or “SD” groups.

^b^Frequency (*n* and %) of the RT‐PCR–positive tests relative to the total number of tests performed in the “Dengue” or “SD” groups.

^c^Frequency (*n* and %) of the specific DENV serotype relative to the total number of RT‐PCR–positive tests.

^d^Frequency (*n* and %) of the RT‐PCR–negative tests relative to the total number of RT‐PCR tests performed in the “Dengue” or “SD” groups.

As expected, the defining manifestations of severity, according to the WHO 2009, were present exclusively in patients with SD. These variables are defined only for the SD group and therefore take structural zero values in the non‐SD dengue group, rather than representing observed absences. The most frequent were plasma leak with shock (42.2%), severe plasma leak without shock (33.4%), organ dysfunction (13.1%), and severe bleeding (2.7%). Two or more severity criteria were observed simultaneously in 21 cases (8.3%), the most common combination being plasma leak with organ dysfunction (*n* = 7).

Of note, among the 251 SD cases in the Huila department, we observed a wide clinical spectrum, ranging from classic plasma leakage and severe bleeding to hyperinflammatory presentations such as HLH, which was identified in 15 of the 114 patients managed in 2019 at the referral center (Hospital Universitario de Neiva) and was associated with a particularly high mortality rate [10].

### 3.3. Viral Serotyping Distribution

Nested RT‐PCR or multiplex RT‐qPCR was performed in 571 patients with confirmed infection to determine the infecting serotype. Virological characterization revealed that NAATs testing for DENV serotypes 1–4 was performed more frequently in SD cases than in dengue cases (*p* < 0.0001) (Table 3). The higher overall testing frequency in SD patients reflects routine prioritization of molecular confirmation in severe cases within the surveillance system. Despite this severity‐based enrichment, the serotyped subgroup was broadly similar to the overall cohort in terms of age distribution, sex, and geographic origin, supporting its use for exploratory description of circulating serotypes (Supporting Table 1).

Overall typing was achieved in 52.2% of the combined NAATs. Late sampling during or after the critical phase, especially in severe cases, likely lowered RT‐PCR typing yield, although this frequency is consistent with previous studies [16, 17]. DENV‐1 was the predominant serotype (82.2% of total typed cases), followed by DENV‐2 (17.8%). No cases of DENV‐3 or DENV‐4 were identified (Table 3). These results are in line with national reports on circulating serotypes in Colombia in 2019 [4].

Among typable samples, a significant difference in serotype distribution was observed. DENV‐1 was predominant in dengue cases, whereas DENV‐2 was more frequently identified in SD cases. Thus, DENV‐2 was enriched considerably in SD patients (56.5%) compared to dengue cases (14.6%) (*p* = 0.0007). The proportion of negative RT‐qPCR tests did not differ significantly between groups (60.0% in SD vs. 46.4% in dengue, *p* = 0.29). This observation suggests an association between the DENV‐2 serotype and disease severity during the 2019 Huila outbreak.

### 3.4. Factors Associated With DENV Infection Severity

Bivariate logistic regression analysis showed that age < 5 years (OR = 3.05; 95% CI: 1.89–4.91) and age between 5 and 15 years (OR = 2.05; 95% CI: 1.30–3.24) were significantly associated with the development of SD, when the reference group was patients aged 16–30 years. Other associated factors included abdominal pain, persistent vomiting, diarrhea, hepatomegaly, somnolence, increased hematocrit, thrombocytopenia < 100,000/mm^3^, hypotension, and fluid accumulation (Table 2). However, the model showed that variables such as diarrhea, vomiting, somnolence, and increased hematocrit had lower ORs (Table 2), indicating that they were influenced by other variables, such as age and sex, a previously reported effect [18].

After adjusting for confounders in the multivariate model, age < 5 years (OR = 4.03; 95% CI: 2.36–6.88), 5–15 years (OR = 1.91; 95% CI: 1.16–3.15) (using patients aged 16–30 years as the reference group), abdominal pain (OR = 3.26; 95% CI: 2.31–4.60), diarrhea (OR = 2.12; 95% CI: 1.55–2.90), persistent vomiting (OR = 1.82; 95% CI: 1.23–2.24), hepatomegaly (OR = 4.78; 95% CI: 3.40–6.72), thrombocytopenia (OR = 7.29; 95% CI: 5.23–10.16), and fluid accumulation (OR = 10.35; 95% CI: 6.01–17.83), maintained statistical significance (Table 4). In summary, the severity of DENV infection was associated with pediatric age and specific clinical and laboratory signs, particularly gastrointestinal, fluid accumulation, and thrombocytopenia. The coherence between the warning signs and progression to severity is consistent with the WHO 2009 criteria for early clinical classification of dengue cases.

**TABLE 4:** The multivariate logistic regression model identified factors associated with SD among patients in the 2019 outbreak in Huila, Colombia.

Condition	Adjusted OR	95% CI	*p* values
Age younger than 5 years	4.03	2.36–6.88	< 0.001
Age 5–15 years	1.91	1.16–3.15	0.012
Persistent vomiting	1.82	1.23–2.24	0.004
Diarrhea	2.12	1.55–2.90	< 0.001
Abdominal pain	3.26	2.31–4.60	< 0.001
Hepatomegaly	4.78	3.40–6.72	< 0.001
Thrombocytopenia < 100,000 cells/mm^3^	7.29	5.23–10.16	< 0.001
Fluid accumulation	10.35	6.01–17.83	< 0.001

*Note:* Age, sex, and comorbidities adjusted ORs are shown.

## 4. Discussion

Studies analyzing factors associated with SD in South America are scarce. In our cohort, pediatric age was an important determinant of severity, with the group younger than 5 years being the most affected, followed by children between 5 and 15 years of age. This association has also been reported in studies in Asia and America, where children presented more frequently with severe forms such as dengue hemorrhagic fever or dengue shock syndrome, in addition to accelerated clinical progression [19–21]. Higher capillary permeability in children, associated with angiogenesis processes and lower endothelial glycocalyx thickness, has been proposed as a pathophysiological explanation [22, 23]. This reinforces the need to prioritize this group in prevention campaigns and hospital triage. However, some studies have identified higher mortality in adults, suggesting that differences in circulating serotypes, pre‐existing immunity, and ethnic factors could influence these patterns [24–26].

A trend toward a higher frequency of severe cases in patients in the subsidized health insurance system was observed, which could reflect barriers in access, timeliness, or quality of care, in line with studies that highlight the influence of social determinants on clinical outcomes [27, 28]. From a health‐systems perspective, this pattern suggests that epidemic preparedness plans in hyperendemic regions should explicitly allocate additional pediatric care capacity and monitoring resources to facilities serving predominantly subsidized‐insurance populations.

The revised WHO 2009 classification has shown good sensitivity for identifying cases progressing to severe forms [29]. Importantly, in line with the WHO 2009 definition, fluid accumulation, hemoconcentration, hypotension, and profound thrombocytopenia in our analysis should be interpreted as clinical markers or components of SD identified at or near the time of classification, rather than as independent early “risk factors” for disease progression. Consistent with this, in this study, symptoms such as abdominal pain, persistent vomiting, and thrombocytopenia < 100,000/mm^3^ were present in more than 60% of severe cases, reaffirming their clinical value [7, 30]. However, atypical presentations of dengue, such as neurological or severe inflammatory syndromes, are not covered by this classification and have been reported, leading to the proposal of an update [31, 32]. In this cohort, 76% of SD cases presented vascular leakage with or without shock, which coincides with Latin American reports but differs from data from Africa and Asia, where severe hemorrhage predominates [33–36]. In recent studies of the 2019 epidemic, we reported a significant number of pediatric cases of hemophagocytic lymphohistiocytosis secondary to subsequent DENV infection, a condition associated with increased mortality and characterized by immune hyperactivation [10]. This supports the need to consider new clinical and immunological markers (such as soluble CD25, ferritin, IL‐6, among others) to complement the case definition of SD in the guidelines.

Although DENV‐1 was the dominant serotype in the epidemic, DENV‐2 was identified more frequently among severe cases. These findings are consistent with regional evidence that the Asian‐American genotype of DENV‐2, circulating in Colombia during the study period, has been associated with large outbreaks and higher severity—particularly among children—in both Colombia and neighboring countries [37, 38]. Prior epidemiological and phylogenetic studies have documented the expansion of this genotype and its temporal relationship with increased incidence of SD since the 1990s in Colombia [39]. Conversely, DENV‐1 is typically linked to milder disease, with severe cases more often observed in adults, reflecting distinctive serotype and age‐related risk profiles reported in Latin America. Structural factors in the envelope and PrM proteins that increase the virulence of the Asian‐American DENV‐2 genotype have been reported in Brazil and, more recently, in India [40–42]. DENV‐3 has also been responsible for a significant number of pediatric SD cases in Central America recently [43], highlighting the effect of geographic variability in viral circulation patterns. Although serotype‐specific inferences are limited by selection bias, these observations underscore the importance of maintaining enhanced serotype surveillance, particularly for DENV‐2, to anticipate periods of increased hospital and intensive care demand.

The high ORs observed must be interpreted in the context of the cross‐sectional study design. The 4.3% prevalence of confirmed SD means these ORs, while valid measures of strong association, may overestimate the relative risk for disease progression from a longitudinal perspective. However, the multivariate modeling confirmed the association of pediatric age, abdominal pain, diarrhea, vomiting, hepatomegaly, thrombocytopenia, and fluid accumulation with SD, which aligns with previous studies [44–46]. These findings reinforce the value of the proposed WHO criteria for early identification of severe cases and support their use to guide clinical decisions, particularly in the pediatric population.

In practical terms, these findings support strengthening triage and referral protocols that prioritize children under 5 years presenting with abdominal pain, vomiting, diarrhea, hepatomegaly, and early thrombocytopenia for closer monitoring, repeated laboratory assessment, and timely referral when needed. The generalizability of these findings to primarily adult populations is limited, and extrapolation of the proposed stratification approach to adult cohorts should be made with caution pending dedicated external validation.

The findings of this study have implications for clinical practice and public health in hyperendemic areas. The identification of early clinical factors allows timely recognition of patients at increased risk of progressing to SD, particularly children. In addition, the finding of a potentially higher proportion of severe cases among patients in the subsidized health care system suggests that inequities in access and the quality of the health care system may influence clinical outcomes, highlighting the need for targeted interventions in vulnerable populations.

This study has several limitations. First, longitudinal clinical follow‐up was not conducted, which prevents us from assessing symptom progression and outcomes. Thus, we cannot determine whether the identified clinical and laboratory signs preceded the onset of severe manifestations or occurred concurrently. Therefore, our findings should not be interpreted as demonstrating causal “risk factors” for progression. Second, the data come from mandatory surveillance registries, which may lead to underreporting and heterogeneity in data quality (particularly clinical symptoms and secondary infection data). Third, virologic typing was performed in a small proportion of cases, limiting the ability to infer more robust associations between serotypes and severity. While the use of multivariate modeling allowed us to adjust for confounding and better estimate the independent associations between factors and SD, in the context of high outcome prevalence and cross‐sectional design, the magnitude of ORs can still be inflated and may lack precision. Finally, the generalizability of our associated factors is influenced by the predominantly pediatric age of our cohort. The strong association with young age is highly relevant for this outbreak but may limit the direct application of these findings to populations where SD more commonly affects adults or the elderly. Future longitudinal studies are warranted to validate these findings in cohorts where temporality and incidence risk ratios can be more accurately established.

In summary, this study identified clinical and laboratory factors associated with SD in a hyperendemic region of Colombia during one of the largest dengue outbreaks, with a remarkable impact on the pediatric population. The evidence generated reinforces the usefulness of the WHO 2009 classification for the identification of severe cases. These findings are relevant for guiding effective clinical interventions and public health strategies in high‐transmission settings.

## 5. Conclusion

The analysis confirmed that clinical and laboratory factors, including childhood, abdominal pain, diarrhea, vomiting, hepatomegaly, thrombocytopenia, and fluid accumulation, are associated with the presentation of SD in the region. Likewise, a trend toward an association between DENV‐2 serotype and greater clinical severity was observed, despite its lower circulation than DENV‐1. These findings support the usefulness of the revised WHO classification for identifying severe cases and highlight the need to strengthen clinical and virological surveillance and to address inequities in access to health services, especially in hyperendemic regions and among vulnerable populations.

## Author Contributions

Conceptualization: Luis F. Oliveros and Carlos F. Narváez.

Data curation: Luis F. Oliveros and Camilo Rivera.

Formal analysis: Luis F. Oliveros and Carlos F. Narváez.

Funding acquisition: Carlos F. Narváez.

Investigation: Luis F. Oliveros, Camilo Eduardo Rivera, María Clemencia Rojas, and Carlos F. Narváez.

Methodology: Luis F. Oliveros and Carlos F. Narváez.

Resources: Carlos F. Narváez.

Supervision: Carlos F. Narváez.

Writing–original draft: Carlos F. Narváez.

Writing–review and editing: Luis F. Oliveros, Camilo Eduardo Rivera, María Clemencia Rojas, and Carlos F. Narváez.

## Funding

This work was funded by the Vicerrectoría de Investigación y Proyección Social, Universidad Surcolombiana (Grant No 3660), and the Sistema General de Regalías del Departamento del Huila (Grant BPIN 2020000100145), both awarded to CFN.

## Ethics Statement

The study was exempt from informed consent requirements because it used anonymized data from SIVIGILA, with approval from the Secretaría de Salud Departamental del Huila (approval code No 2019CS035391‐1) and the Comité de bioética of the Facultad de Ciencias de la Salud, Universidad Surcolombiana, Neiva (approval code No 007–2019).

## Conflicts of Interest

The authors declare no conflicts of interest.

## Supporting Information

Additional supporting information can be found online in the Supporting Information section.

## Supporting information


**Supporting Information** Supporting Table 1. This supplemental material describes the epidemiological characteristics and clinical classification of patients with confirmed dengue, with or without RT‐qPCR serotyping.

## Data Availability

Deidentified data underlying the findings of this study are available from the corresponding author upon reasonable request. The data are not publicly available because they contain sensitive clinical information and are subject to institutional restrictions.
