# Supplementary material for: Factors Associated With Severe Dengue During the 2019 Outbreak in Southern Colombia: Implications for Hyperendemic Regions
Source: J Trop Med. 2026 Sep 22;2026:2084096. doi: 10.1155/jotm/2084096 (PMC13598374; doi:10.1155/jotm/2084096)
Supplement: Supplementary file 1 [file JOTM-2026-2084096-s001.docx]

**Supplemental Table 1.** Characteristics of patients RT-qPCR serotyped and non-serotyped.

| **Characteristic** | **Serotyped DENV-infected**  **(n = 571)** | **Non-serotyped DENV-infected**  **(n = 5,174)** | ***p-value*** |
| --- | --- | --- | --- |
| Age, median (range) | 13 (1 - 78) | 15 (1 - 90) | 0.3223^a^ |
| Female/male | 299/272 | 2,635/2,539 | 0.5169^b^ |
| Neiva/others | 331/240 | 3,025/2,149 | 0.8229^b^ |
| Dengue/SD cases | 513/58 | 4,981/193 | <0.001^b^ |
| Ratio of RT-PCR tests (+/- results)* | 0.9 (55/60) | - | 0.345^b^ |
| Ratio of RT-qPCR tests (+/- results)* | 1.1 (243/213) | - |  |

^a^U-Mann-Whitney and ^b^chi-square statistical tests were used.* Separate typing yields for each NAATs.
